# Development of Co-Amorphous Systems for Inhalation Therapy—Part 1: From Model Prediction to Clinical Success †

**DOI:** 10.3390/pharmaceutics17070922

**Published:** 2025-07-16

**Authors:** Eleonore Fröhlich, Aurora Bordoni, Nila Mohsenzada, Stefan Mitsche, Hartmuth Schröttner, Sarah Zellnitz-Neugebauer

**Affiliations:** 1 Research Center Pharmaceutical Engineering, Inffeldgasse 13, 8010 Graz, Austria; eleonore.froehlich@medunigraz.at (E.F.); nila.mohsenzada@gmx.at (N.M.); 2 Center for Medical Research, Medical University of Graz, Stiftingtalstr 24, 8010 Graz, Austria; 3 Institute of Electron Microscopy and Nanoanalysis (FELMI), Graz University of Technology, Steyrergasse 17, 8010 Graz, Austria; stefan.mitsche@felmi-zfe.at (S.M.); hartmuth.schroettner@felmi-zfe.at (H.S.); 4 Graz Centre for Electron Microscopy (ZFE), Steyrergasse 17, 8010 Graz, Austria

**Keywords:** co-amorphous, machine learning, inhalation, tuberculosis, ethambutol, rifampicin

## Abstract

**Background/Objectives**: The integration of machine learning (ML) and artificial intelligence (AI) has revolutionized the pharmaceutical industry by improving drug discovery, development and manufacturing processes. Based on literature data, an ML model was developed by our group to predict the formation of binary co-amorphous systems (COAMSs) for inhalation therapy. The model’s ability to develop a dry powder formulation with the necessary properties for a predicted co-amorphous combination was evaluated. **Methods**: An extended experimental validation of the ML model by co-milling and X-ray diffraction analysis for 18 API-API (active pharmaceutical ingredient) combinations is presented. Additionally, one COAMS of rifampicin (RIF) and ethambutol (ETH), two first-line tuberculosis (TB) drugs are developed further for inhalation therapy. **Results**: The ML model has shown an accuracy of 79% in predicting suitable combinations for 35 APIs used in inhalation therapy; experimental accuracy was demonstrated to be 72%. The study confirmed the successful development of stable COAMSs of RIF-ETH either via spray-drying or co-milling. In particular, the milled COAMSs showed better aerosolization properties (higher ED and FPF with lower standard deviation). Further, RIF-ETH COAMSs show much more reproducible results in terms of drug quantity dissolved over time. **Conclusions**: ML has been shown to be a suitable tool to predict COAMSs that can be developed for TB treatment by inhalation to save time and cost during the experimental screening phase.

## 1. Introduction

The integration of Machine Learning (ML) and Artificial Intelligence (AI) into the pharmaceutical industry has been transformative, enabling breakthroughs across various phases of drug discovery, development, and manufacturing [1]. These advanced technologies leverage vast amounts of biological, chemical, and clinical data to make predictions, identify patterns, and optimize processes that would be virtually impossible to achieve using traditional methods [2]. The convergence of AI and ML has revolutionized the industry’s approach to drug discovery and design [3,4], clinical trials [5], personalized medicine [6], and even regulatory processes [7]. In the past, the pharmaceutical industry relied heavily on experimental trial-and-error methods to uncover potential drug candidates and validate their efficacy. However, with the rise of ML and AI, predictive models are now used to simulate biological systems, forecast drug interactions, and estimate the probability of clinical success. This shift from purely empirical experiments to data-driven predictions is arguably one of the most significant tends in pharmaceutical innovation today. AI-powered platforms are now able to quickly sift through vast chemical libraries, identify promising drug candidates and predict potential adverse effects even before the physical testing phase. Artificial neural networks (e.g., Chem software) are used in controlled-release tablet formulation, hard-capsule shell formation, solid dispersions, pellets, and micro- and nanoparticles [8].

The collaboration between advanced algorithms and experimental validation remains essential to ensure that AI- and ML-driven predictions translate effectively into real-world therapeutic solutions. So far, current AI- or ML-based approaches are not a substitute for traditional experimental methods; however, AI and ML can provide predictions based on the data available and potentially limit and facilitate experimental efforts, as the predicted outcome must then be validated and interpreted by human researchers [9]. The integration of AI and ML with traditional experimental methods can enhance pharmaceutical processes like drug discovery processes [4,10], accelerate the development of new medications [3,11], and optimize and control pharmaceutical unit operations during manufacturing [12,13]. Especially for pulmonary drug delivery systems, where the testing in laboratory animals is less predictive than for other administration routes, in silico tools may be very useful [14]. Treatments with more than one drug are common in the treatment of respiratory diseases and combined administration will increase patient compliance. However, the generation of such formulations may pose problems.

For this reason, we have developed a simple predictive ML tool for the formation of co-amorphous systems (COAMSs) to support scientists and enable rapid screening and minimize laboratory effort, time and cost [15]. A molecular descriptor-based ML model for predicting the potential of binary drug combinations to form co-amorphous systems was built based on available literature data (generation of a literature database). In contrast to previously reported predictive models, input data from four classes of COAMS (active pharmaceutical ingredient (API)-API (1), API—amino acid (2) API—organic acid (3) and API—other substance (4)), making the model relatively universally applicable. The accuracy of the generated ML model was 79%. Predictions were made for 35 active pharmaceutical ingredients (APIs) used in inhalation therapy [15]. The inhalation route was taken as in typical lung diseases, namely asthma, chronic obstructive pulmonary diseases (COPDs) and tuberculosis (TB), it is common to apply multiple drugs and combinations over a longer period [16,17,18,19]. COAMSs for inhalation therapy are suggested to improve patient compliance by reducing the number of medications to take and decrease the drug dose variability while administering combination products to patients. Further, COAMSs have advantageous properties, such as improved solubility, dissolution, and stability [20]. They are defined as homogeneous single-phase systems where typically an API is combined with a co-former (low-molecular-weight compound) and the system is stabilized. A co-former can either be another API or an excipient (e.g., amino acid, organic acid). The selection of suitable co-formers is crucial for successful co-formability and a lack of systematic, predictive, and computational methods for this selection has been identified [21]. An overview on predictive models available for co-former selection can be found in our previous paper [15].

So far only three systems have been tested experimentally and published together with model building [15]. In the present work, a further 15 systems were selected for in-depth model validation. Further, one relevant therapeutic system was selected and developed as a dry powder for inhalation therapy. To demonstrate the practical value of the model, a combination of two first-line drugs for TB treatment, rifampicin (RIF) and ethambutol (ETH), was selected due to the global importance of TB and the lack of inhaled formulations for TB treatment. TB is one of the major global health burdens, caused by Mycobacterium tuberculosis and mainly affecting the lungs, but it can also spread to other parts of the body [22]. Before the COVID-19 pandemic, TB was the most prevalent infectious disease worldwide [23]. To date, the standard therapy is still oral or parenteral, involving various antibiotics over a long period of time. However, there is a big research focus on TB treatment via inhalation [24,25,26,27]. Delivering TB drugs via inhalation could lower systemic doses while achieving higher lung doses (site of action and primary location of disease) and efficacy [28]. Although tuberculostatic drugs for inhalation were tested in preclinical studies, few formulations, e.g., dry powder amikacin (NCT04249531) and nebulized rifampicin (NCT06041919), have been evaluated in clinical trials, and no formulation has reached the market yet.

Our goal is to develop an innovative drug–drug COAMS for inhalation therapy of TB. Therefore, an extended validation of the predictive ML tool to predict COAMS (developed by our group and published previously in Pharmaceutics) was first conducted. Based on the results, a promising co-amorphous combination of RIF-ETH was selected further and developed as powder for inhalation therapy. For efficient delivery to the lung, particles should be 1–5 µm [29]. Therefore, particle size, solid state, morphology, aerosolization performance, and dissolution of the developed dry powder formation were determined.

## 2. Material and Methods

### 2.1. Materials

The following active ingredients were used for model validation: salbutamol sulfate (SBS) (Fagron GmbH, Glinde, Germany), glycopyrronium bromide (GB) (kindly donated from Chiesi Pharmaceuticals, Parma, Italy), mometasone fuorate (MOM), bambuterol HCl (BAM) (both from Shenzhen Nexconn Pharmatechs Ltd., Shenzhen, China), isoniazid (ISO), streptomycin sulfate (STR), ethambutol (ETH), pyrazinamide (PYR), budesonide (all purchased from TCI Deutschland GmbH, Eschborn, Germany) and rifampicin (RIF) (Olon Active Pharmaceutical Ingredients, Mumbai, India).

Inhalac 500 (Meggle GmbH, Wasserburg, Germany), Magnesium Stearate ((MgSt) (Sigma-Aldrich, Darmstadt, Germany)) and Leucin (Merck KGaA, Darmstadt, Germany) were used as fine excipients.

Solvents were used for analytics (acetic acid, acetonitrile, methanol) and spray-drying (absolute Ethanol). Absolute ethanol was purchased from Lactan Chemikalien and Laborgeraete Vertriebsgesellschaft m.b.H & Co. KG (Graz, Austria).

For the preparation of simulated lung fluid (SLF), Gibco™ PBS, pH 7.4 buffer (Fisher Scientific GmbH, Vienna, Austria) and 1,2-Dipalmitoyl-sn-glycero-3-phosphocholine (DPPC) (TCI Deutschland GmbH, Eschborn, Germany) were used.

For aerosolization tests hard-gelatin Coni-Snap^®^ size 3 capsules provided by Capsugel (Köln, Germany) and a capsule-based inhaler, the Cyclohaler^®^ (PB Pharma GmbH, Meerbusch, Germany), were used.

### 2.2. Model Validation

The ML model predicts the potential formation of binary COAMSs in a molar ratio of 1:1. For simplicity, this common molar ratio of 1:1 was chosen as it was the most commonly used in the literature when testing COAMSs, even though it may not be therapeutically relevant or effective. The preparation of binary COAMSs in therapeutically relevant ratios will be addressed in a follow-up study (Development of Co-Amorphous Systems for Inhalation Therapy—Part 2). For extended model validation, 15 additional systems (11 positively predicted and 4 negatively predicted) were selected based on the availability of APIs in house, the cost of the APIs, considerations of therapeutically relevant combinations, and the selection of positive and negative examples. Selected systems are shown in Table 1. The two respective APIs were weighed together in a molar ratio of 1:1 (total of 1 g); placed in a 50 mL metal mixing cylinder together with a stainless-steel ball (20 mm diameter), and then milled for 10 min at 25 Hz in a cryo-mill (Retsch GmbH, Haan, Germany) operated without nitrogen. After milling, powder diffraction measurements were conducted (see Section 2.4). As for initial model validation, milling (an easy and common kinetic method described for co-amorphization) was chosen for extended model validation as well.

### 2.3. Preparation of COAMS

For further tests, a combination of two TB first-line drugs was selected, RIF and ETH. For preparation of the COAMSs of RIF-ETH, co-milling and spray-drying was chosen. Co-milling and spray-drying are both common methods used for co-amorphization. For co-milling, the two APIs were placed in a molar ratio of 1:1 (total amount 1 g (252 mg ETH and 748 mg RIF)) in a 50 mL mixing cylinder together with a stainless-steel ball (20 mm diameter) and then milled for 10 min at 25 Hz in a cryo-mill (Retsch GmbH, Germany) operated without nitrogen, followed by a jet-milling step to obtain a size suitable for inhalation (<5 µm) (Hosokawa Spiral Airjetmill 50 AS; same settings as described below for the PM).

For spray-drying, the 2 APIs (molar ratio 1:1) were dissolved in a solution of ethanol:water (80:20) with a solid concentration of 4% and spray-dried using the BUCHI Mini Spray Dryer B-290 (Buchi Labortechnik AG, Flawil, Switzerland) in a closed-loop configuration. An inlet temperature of 85 °C, an airflow rate of 1052 L/h, a spray rate of 9% (2 mL/min) and a nozzle cap 1.4 mm were used. Particles were collected in a cyclone.

As a reference, a physical mixture (PM) of micronized crystalline RIF and crystalline ETH (molar ratio 1:1, total 5 g)) was prepared in a tumble blender (T2F Turbula^®^, Willy A. Bachofen AG Maschinenfabrik, Muttenz, Switzerland) at 32 rpm for 30 min. Micronization of RIF and ETH was performed with a jet mill (Hosokawa Spiral Airjetmill 50 AS). The powder was slowly manually fed and an inlet pressure of 6 bar and a milling pressure of 3 bar were set.

All samples were stored dry in a desiccator over silica gel at room temperature (25 ± 2 °C) after preparation and only removed for a different analysis for the minimum time required.

### 2.4. Particle Characterization

#### 2.4.1. Particle Size Measurements—LiteSizer

The particle size was measured via dynamic light scattering with a Litesizer 500 (Anton Paar GmbH, Graz, Austria) equipped with a 658 nm laser. Prior to the measurements, saturated solutions were prepared, in which the respective powders for analysis were suspended afterwards. For starting material analysis, solutions were saturated with either RIF or ETH, and for COAMS RIF-ETH analysis, solutions were saturated with RF and ETH. The refractive index (RI) for rifampicin was 1.613 and for ethambutol it was 1.461. An RI of 1.3303 was applied for MQ-water. Each sample was measured three times at 25 °C using an angle of 175° (back scatter) and an equilibrium time of 30 s. For each measurement, ten runs were applied using a measurement duration of 5 s per run. By means of PCS, the mean hydrodynamic diameter (intensity based), which is calculated from the signal intensity and the polydispersity index (PdI; a measure of the width of the droplet size distribution) were determined.

#### 2.4.2. Solid-State—Powder X-Ray Diffraction (XRD) Measurements

Powder diffraction using X-ray was used to determine the solid-state of the co-processed samples and to verify co-amorphization. The starting materials (individual APIs before milling) and the co-processed samples were analyzed via XRD (maximum time from milling to analysis was around 1 h) immediately after preparation. Analysis was carried out with a Siemens D5005 (in Bragg–Brentano geometry) equipped with a Cu-Anode (λ = 1.54186 A) and operated at 40 kV and 40 mA. The measurements were performed in a 2 Theta range between 4° and 40°, with a step size of 0.04° and a time per step of 2 s. A scintillator detector was used for counting the X-rays. In order to have a also reference for the further-developed RIF-ETH COAMS, a physical mixture of RIF-ETH was analyzed via XRD.

#### 2.4.3. Particle Morphology—SEM Images

Scanning electron microscopy (SEM) (Zeiss Ultra 55, Zeiss, Oberkochen, Germany) was used to analyze the surface morphology of pure APIs (RIF, ETH), the co-processed RIF-ETH samples and the PM of RIF-ETH. All samples were gold-palladium sputtered prior to analysis and the instrument was operated at 5 kV.

### 2.5. Formulation Preparation

The co-processed RIF-ETH combinations and the PM were filled manually as carrier-free drug-only formulations in size 3 hard-gelatin capsules. Capsule fill weight was 25 ± 1 mg. Typically, in addition to special technologies [30], carrier-based formulations, where the small API particles are attached to a larger carrier particle (50–200 µm; most commonly lactose monohydrate), are used within DPIs. The carrier and API should form a stable formulation that is easier to handle, transport and dose; however, during inhalation, the API must again separate from the carrier as only small particles below 5 µm can reach the lung [31].

Additionally, adhesive mixtures of co-milled COAMS samples with different ternary agents were prepared in a TC2 tumble blender (Willy A. Bachofen Maschinenfabrik, Muttenz, Switzerland) in closable stainless-steel vessels (inner diameter 49 mm, height 40 mm, filling volume about 40%). The batch size was 5 g. For each mixture, the ternary agent was layered between the API using the sandwich method and blended for 60 min at 32 rpm. The ternary agents used were leucine (5%), MgSt (0.5%) and fine lactose (10%). Ternary agents are typically used with DPI formulations to facilitate drug deagglomeration and dispersion and drug detachment from the carrier during the inhalation process [32].

### 2.6. Analytics

RIF content in the samples was determined using high-performance liquid chromatography (HPLC) with the Acquity Arc system (Waters Corp., Milford, CT, USA), coupled with a photodiode array (PDA) detector. Quantification was performed at a wavelength of 236 nm. The mobile phase consisted of 55% phosphate buffer (30 mM, pH 4.0) and 45% acetonitrile (ACN), under isocratic elution at a flow rate of 0.6 mL/min. The stationary phase was a SunFire C18 column (3.5 μm, 4.6 × 150 mm). The column temperature was maintained at 25 °C, and a 5 µL aliquot of the sample solution was injected. Each sample was analyzed in duplicate. Method linearity was confirmed over the concentration range of 1–300 µg/mL. The estimated limits of detection (LODs) and quantification (LOQs) were 0.02 µg/mL and 0.05 µg/mL, respectively.

ETH content was measured using an Ultra Performance Liquid Chromatography (UPLC) H-Class system (Waters Corp., Milford, CT, USA), coupled with a single quadrupole mass detector (QDa). The mass spectrometer operated in selected ion recording (SIR) mode with positive ionization, targeting a mass of 205 Da within a retention time window of 0–5 min. Instrument parameters included a cone voltage of 5 V, a probe temperature of 400 °C, and a capillary voltage of 0.8 kV. The mobile phase consisted of 50% 0.1% triethylamine (TEA) in water, adjusted to pH 7.0, and 50% ACN. Chromatographic separation was performed on an Agilent Zorbax SB-CN column (2.1 × 150 mm, 5 μm) under isocratic conditions at a flow rate of 0.7 mL/min. The column temperature was set to 30 °C, with a 1 µL injection volume. Each sample was analyzed in duplicate. The linearity of the method was validated over the range of 1–200 µg/mL. The estimated LOD and LOQ were 0.03 µg/mL and 0.1 µg/mL, respectively.

### 2.7. API Distribution/API Content

The uniformity of API content within the PM, the spray-dried RIF-ETH COAMS and the cryo-milled RIF-ETH COAMS were determined by taking 10 samples of around 5 mg of the respective formulation, and analyzing it via HPLC (Section 2.6: Analytics) for drug content. Samples for ETH content analysis were dissolved in 25 mL diluent (respective HPLC mobile phase) and samples for RIF content analysis were dissolved in 25 mL diluent (respective HLC mobile phase). The mean drug content is expressed by the mean drug content of the 10 samples in % and the mixing homogeneity is expressed by the relative standard deviation of the mean drug content within the 10 samples.

### 2.8. Aerosolization Performance

The aerodynamic performance of the blends was assessed using the Fast Screening Impactor (FSI) (Copley Scientific, Nottingham, UK) together with the Cyclohaler^®^, a capsule-based low-resistance inhaler. Capsules used were manually filled with a 30 ± 2 mg drug-only formulation. The methodology followed the European Pharmacopoeia (preparations for inhalation: aerodynamic assessment of fine particles, Ph. Eur., 7.0). The FSI divides the aerosol into two fractions; particles with an aerodynamic diameter > 5 µm are collected inside the induction port and the coarse fraction collector (CFC), and particles with an aerodynamic diameter < 5 µm are collected in the fine fraction collector (FFC) on a glass fiber filter. During the experiments an air flow rate of 60 L/min was set to ensure a pressure drop of 4 kPa and the appropriate CFC insert was used for a flow rate of 60 L/min. The flow was applied for 4 s to ensure that 4 L of air were drawn via the mouthpiece over the inhaler. The drug content in each part of the impactor was quantified using a validated analytical method (see Section 2.6) and each blend was analyzed in triplicate. In order to compare the performance of the different formulations, the fine particle fraction (FPF) and the emitted fraction (EF) were chosen. The EF (%) indicates the percentage of API found in the whole impactor (induction port CFC and FFC) related to the target dose. The FPF gives the percentage of API particles exhibiting an aerodynamic diameter of <5 µm. The FPF (%) is calculated as the ratio of fine particle mass (FPM) (mass of particles below 5 µm, equal the mass on the FFC) and emitted dose (mass emitted per FSI experiment). Each formulation was tested in triplicate.

### 2.9. Dissolution

Dissolution experiments were performed in simulated lung fluid (SLF) over 24 h. SLF was prepared by dissolving 28 mg DPPC in 2.1537 mL ethanol. The DPPC solution was then added slowly and carefully to 140 mL of preheated PBS buffer (approximately 37 °C) while stirring. The slightly turbid solution then was put into an ultra-sonic bath until the solution was clear (approximately 20 min at 37 °C).

For the dissolution tests, FSI experiments were performed as described above and the filter from the FFC that collects particles below 5 µm was used. The filters were placed into a crystallizing dish (volume 250 mL). Afterwards, 55 mL of SLF was added and the crystallizing dish was placed onto a laboratory shaker at 37 °C and 60 rpm. To date, different methods and setups for the dissolution testing of inhalable products have been described in the literature, but a standardized method has not been proposed yet [33]. The setup is a membrane-based system, similar to both the Franz diffusion cell and the Transwell system. The design was modified based on the sensitivity of HPLC detection and the size of the FSI membrane. The sampling conditions and protocol adhered to the standards of the United States Pharmacopeia (USP) and the National Formulary (NF) [34]. The sampling was performed immediately (time 0) and after 5, 10, 20, 40, 60, 120 and 180 min. Each time, 500 µL was taken and the total dissolution volume was kept constant, by adding 500 µL SLF every time a sample was taken. Experiments were performed in triplicate. Samples were filled into HPLC vials and analyzed for drug content via HPLC.

The cumulative dissolved amount was calculated according to the following equation:

$$Q_{cum}=C_{1}\times v+C_{2}\times v\times \left(1-\frac{v}{V}\right)+\;C_{3}\times v\times {\left(1-\frac{v}{V}\right)}^{2}+\;\;C_{4}\times v\times {\left(1-\frac{v}{V}\right)}^{3}+\;C_{5}\times v\times {\left(1-\frac{v}{V}\right)}^{4}$$

where *Q_cum_* is the cumulative amount of drug dissolved, *C* the drug concentrations at the five time points, *v* the sample volume, and V the total volume.

According to the USP <711>, the dissolved amount is indicated in “%” of label claims. Maximum cumulative release (e.g., dissolved after 180 min), or maximum solubility if the compound is poorly soluble, can also be set as 100%. The indication of the absolute permeated amount (in µg) can also be used [35].

For inhalation formulations, the determination of the 100% value for dissolution is not straightforward, as capsules are filled manually and show slight variations, and only a certain % of initial API amount reaches the filter (FPF). Dissolution graphs have shown that a plateau of API concentration was reached after a certain time (around 10 min); therefore, the amount of API dissolved after 180 min was considered as a value of 100%.

Samples were stored with light protection and frozen to prevent degradation prior to HPLC analysis.

### 2.10. Stability Study

To assess the stability of the COAMS, the samples were kept in desiccators over silica gel at ambient temperature (25 ± 3 °C). Initially and after 1 day, 3 days, 10 days and 365 days, XRD measurements were conducted in order to obtain information on the amorphous and crystalline state.

### 2.11. Statistical Analysis

Differences between the three formulations (RIF-ETH PM, RIF-ETH CM and RIF-EH SD) regarding key parameters like D50, FPF and ED were statistically analyzed using a one-way ANOVA Test. Once a significant difference was found using ANOVA, a two-tail *t*-Test of means (two samples, assuming unequal variances) was performed between pairs of the three formulations to determine which formulations differed significantly. Microsoft EXCEL software was used for the ANOVA and *t*-test and the significance level was set at 0.05 (*p* = 0. 05) for both tests.

## 3. Results and Discussion

### 3.1. Detailed Model Validation

Based on literature data, an ML model was built to predict the formation of binary COAMS [15]. The decision tree model is based on gradient boosting method and was developed on a training dataset of 254 systems. A careful selection of molecular descriptors (Table S1: List of selected molecular descriptors) was used to obtain a numerical description of the systems. For each substance pair (COAMS or non-COAMS), the absolute differences between the API and co-former molecular descriptor values were computed, yielding 29 input features. An additional four features captured hydrogen bonding and acid/base interactions, focusing on donor–acceptor relationships. In total, 34 input features were generated. The average values of input features were compared between COAMS and non-COAMS systems. Features with smaller differences in COAMS, such as relative hydrophobic surface area (RASA), molecular framework ratio (fmF), and topological shape index (Topo(Shape)), indicated that structural similarity favored COAMS formation. Conversely, larger differences in features like rotatable bond count (nRot), hydrogen bond donors (nH), and basic groups (nBase) were associated with COAMS, suggesting that dissimilarity in these parameters may promote their formation. To increase the robustness and generalizability of the ML model, an ensemble learning approach in combination with cross-validation was chosen. Therefore, 50 independently trained models were generated using cross-validation after hyperparameter tuning, and their predictions were averaged to obtain the final result. For the 50 models a random 85%/15% split for training and individual validation data was performed. Additionally, the model was validated with 19 completely separate and unseen data. The model accuracy was 79%, and after model building (training and validation), predictions were made for 35 drugs used in inhalation therapy as an input factor. Validation data for three systems (two positively predicted and one negatively predicted) have already been published with the model [15]. Additionally, 15 predicted API-API pairs (10 positively and 5 negatively predicted, a total of 18) were tested experimentally. Besides selection criterial mentioned in Section 2, examples with more or less certain prediction values were chosen; either very high (close to 1) or very low (close to 0). The model gives for any possible API-API combination (out of the 35 Input APIs) a predicted score between 0 and 1. A high predicted score (close to 1) means a high certainty to form a COAMS. The ability of the generated ML model to predict the formation of COAMS in new combinations of substances is limited by how similar they are to the training dataset. Results for API combinations with molecular descriptors that differ significantly from those in the training data are less reliable. As a result, the distance of all new combinations from the training dataset was calculated and considered. Distance values from 0 to 1600 were reported by the model and only systems with values below 450 were considered. The respective API pairs were co-milled together and the amorphous state verified via XRD analysis. The API pairs, together with prediction, distance, therapeutic relevance and XRD results, are summarized in Table 1. All XRD graphs can be found in the Supplementary Material (Figure S1A–R). XRD Graphs showed that MOM-BAM, SBS-RIF, RIF-GB, STR-GB, RIF-ETH, MOM-SBS, MOM-GB, RIF-PYR, BUD-GB and SBS-GB that were co-amorphous as predicted (predicted scores 0.9 to 1), and ISO-PYR, ETH-PYR and ISO-ETH were still crystalline, although they were predicted as co-amorphous (predicted scores 1.0, 0.96 and 0.90, respectively). For the negative examples, BAM-PYR and BAM-ISO were still crystalline after milling as predicted; however, STR-BUD and MOM-STR became amorphous, although they were predicted as crystalline.

Summing up, co-milling experiments confirmed that the predictions of 10 out of 13 positively predicted COAMSs and of 3 out of 5 negatively predicted non-COAMSs were correct, resulting in an experimental accuracy of 72%.

The discrepancies between model predictions and experimental outcomes—specifically, three false positives (ISO-PYR, ETH-PYR, ISO-ETH) and two false negatives (STR-BUD, MOM-STR)—highlight key limitations and areas for improvement in our modeling approach. The false positives may stem from multiple factors. First, processing conditions such as milling time, temperature, and humidity, which significantly influence co-amorphous formation, were not included in the model due to inconsistent reporting in the literature. Second, the molecular descriptors used may not fully capture specific intermolecular interactions, such as hydrogen bonding networks, or the thermodynamic and kinetic aspects of solid-state behavior. Notably, all three falsely predicted positive cases involve small, hydrophilic molecules with multiple hydrogen bond donors and acceptors, suggesting that their intrinsic miscibility and amorphization potential may not be fully represented by the current descriptor set. Further, similarity to the training data plays a key role; compound combinations that are underrepresented or structurally distinct from the training set may be more prone to misclassification. Although, this was not the case for the falsely positive predicted combinations (Distance values < 60). these cases may still lie outside the optimal domain of applicability.

In addition, formulation-specific factors such as stoichiometric ratio may contribute to discrepancies. While a 1:1 molar ratio was used consistently in our experiments, some studies have shown improved co-amorphization at ratios between 40:60 and 60:40, particularly for drug–drug combinations [36]. Therefore, it is possible that a different ratio could result in amorphous systems for the PYR/ISO, PYR/ETH, and ETH/ISO combinations. Furthermore, the high hydrophobicity of ethambutol dihydrochloride may be the reason for the crystalline mixtures with PYR and ISO in co-amorphous systems containing ETH [37]. Conversely, the two false negative predictions (STR-BUD, MOM-STR) involve combinations with streptomycin, a large, highly polar molecule that was already amorphous in its initial state. As a result, the outcome cannot be classified as crystalline, even if co-amorphization did not occur in the classical sense. Streptomycin can exist in an amorphous or crystalline form; in particular, the sulfate can exist in an amorphous form [38], which introduces ambiguity in the interpretation of experimental results and highlights the importance of considering the initial physical state of the components in model development.

Based on the predictions made and model validation experiments, one therapeutically relevant system of two first-line drugs for TB treatment (RIF and ETH) was selected and developed further as powder for inhalation. Particle properties relevant to DPIs were not considered for model validation but were considered for the selected model system and are analyzed and discussed below.

### 3.2. Particle Characteristics

Model validation experiments showed that co-milling is able to generate RIF-ETH COAMSs. However, in order to obtain a particle size below 5 µm suitable for inhalation, micronization was performed, resulting in particles of 2.78 ± 0.95 µm. Besides co-milling, spray-drying of RIF-ETH was performed to generate particles in the inhalable size range. Spray-drying offers the advantage of also being able to generate COAMSs [39] and simultaneously control particle size by the selection of process parameters [40].

**Figure 1:**
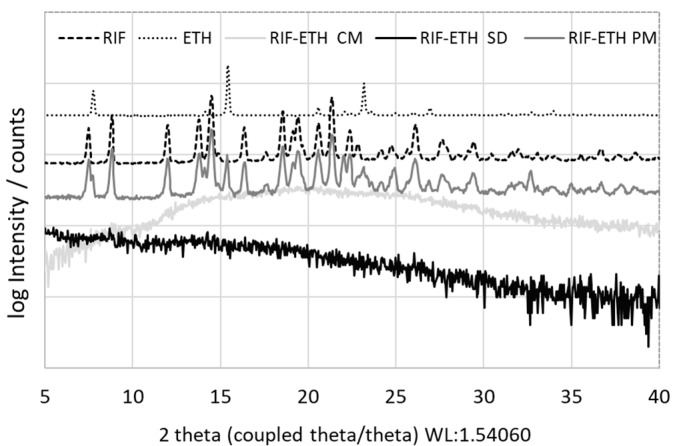
XRD pattern of the starting materials (jet milled RIF and ETH), the physical mixture of RIF and ETH (RIF-ETH PM), the co-amorphous milled (RIF-ETH CM) and spray-dried (RIF-ETH SD) RIF-ETH formulation.

To proof amorphization after milling and spray-drying, XRD measurements of starting materials (RIF, ETH), the physical mixture and milled as well as spray-dried RIF-ETH formulations were performed. Results in Figure 1 show the crystalline nature of RIF, indicated by several characteristic peaks at 7.36°, 8.70°, 13.66°, 14.38°, and 21.26° [41]. On the other hand, ETH has three main peaks at 7.60°, 15.30° and 23.0° that confirm its crystalline nature [42]. The physical mixture of RIF-ETHs shows characteristic peaks of both APIs, confirming that both APIs are still present in their crystalline form. For RIF-ETH milled and spray-dried, no Bragg peaks were observed, but only the amorphous halo, typical for amorphous samples, was visible. This indicated successful co-amorphization.

**Figure 2:**
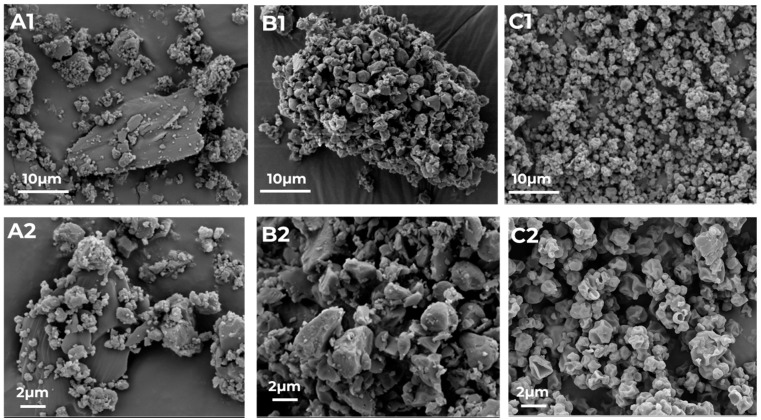
SEM images of the physical mixture of RIF and ETH ((RIF-ETH PM), (**A1**,**A2**)), and the co-amorphous milled ((RIF-ETH CM), (**B1**,**B2**)) and spray-dried ((RIF-ETH SD), (**C1**,**C2**)) RIF-ETH formulation at 2 magnifications (image width 57.16 µm and 22.87 µm).

Particle size analysis confirmed that spray-dried as well as milled particles and the PM of micronized crystalline RIF and ETH are in a size range suitable for inhalation (Table 2). The spray-dried particles are slightly smaller compared to the milled particles and based on the SPAN values, both particles are more or less evenly distributed. A one-way ANOVA was performed to compare the mean D50 between the three formulations (CM, SD and PM) and no significant difference was found (*p* = 0.15). Although the SPAN of the PM is only slightly higher, SEM images in Figure 2 indicate that co-amorphous samples are much more uniform regarding their particle size and appearance.

The PM is not completely homogeneous as still particles of the 2 APIs can be distinguished. (SEM images of pure RIF and ETH can be found in the Supplementary Information, Figure S2). ETH exhibits more plate-like elongated particles while RIF shows smaller and more round particles. In Figure 2A1,A2, larger plate-like ETH particles with smaller RIF and ETH particles on the surface are visualized.

Both co-amorphous particles are more homogeneous compared to the PM. However, the milled sample still shows the different particle shapes of the two starting APIs (Figure 2B1,B2), while in the spray-dried sample all particles have the same uniform wrinkled surface (Figure 2C1,C2).

### 3.3. COAMS Stability

Stability for both systems was measured and is guaranteed for 1 year, considering storage conditions at ambient temperature (25 ± 3 °C) and in a dessicator over silica gel. After only 1 year, an amorphous halo is still present for the spray-dried as well as the milled formulation and no peaks indicating recrystallization could be detected (Supplementary Material, Figure S3).

### 3.4. Particle Performance

The spray-dried and milled co-amorphous formulations were tested regarding their aerosolization properties. The doses required for TB treatment orally (e.g., rifampicin 450–600 mg and ethambutol 800–2000 mg) are rather high compared to typical doses for most APIs for inhalation therapy of asthma and COPD (e.g., salbutamol sulfate 200 to 1800 µg, formoterol 6–12 µg, budesonide 200–800 µg). As the guideline for effective inhalable dose is 10 times lower than the oral dose [43], doses are still comparably high and consequently no carrier was used and a carrier-free drug-only formulation was tested. Carrier-free formulations are the preferred option when targeting high drug dosages through DPIs [44], as in our case. RIF-ETH SD and RIF-ETH CM were compared with a physical mixture of crystalline RIF and ETH.

RIF and ETH mean drug content and mixing homogeneity results in Table 3 show that overall the drug content within the physical mixture shows the highest variations (104% RIF and only 81% ETH). The physical mixture also shows the poorest mixing homogeneity (10.2% for ETH and 6% for RIF). Typically for inhalation applications, a mixing homogeneity of less than 5% is considered homogenous and acceptable [45]. For the co-amorphous SD and CM formulations, the mixing homogeneity is below 5% and consequently the formulations can be considered homogenous. For single-dose medicinal products, the assay is usually expressed as mass per dosage unit and a deviation of 5% is acceptable [46]. Here we evaluate the drug content within the blend and, except for the PM and ETH within the SD formulation, the drug assay is within the required range.

**Figure 3:**
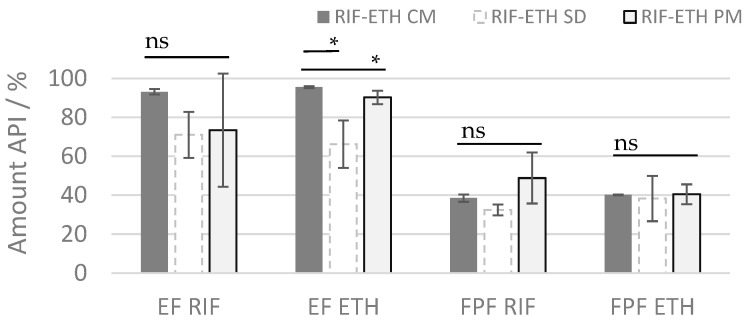
ED and FPF or RIF and ETH for the 3 different formulations (ETH RIF PM, ETH-RIF CM, ETH-RIF SD) (mean ± SD, *n* = 3), ns: not significant; significant at * *p* ≤ 0.05.

Aerodynamic performance results (Table 4 and Table 5 and Figure 3) showed a superior behavior of the co-milled COAMS compared to the spray-dried COAMS and the physical mixture (PM). The amount of drug released from the inhaler (ED RIF = 93.1% and ED ETH = 95.9%) and the amount of drug reaching the lungs (FPF RIF = 38.5%, and FPF ETH = 40.2%) is higher compared to the other formulations. This is also reflected in a significantly higher FPM (for RIF and ETH) of the co-milled formulation compared to the spray-dried formulation (*p* ≤ 0.05). The rather low ED for the spray-dried COAMS (71.0% for RIF and 66% for ETH) indicates that a lot of powder remains in the inhaler or capsule, which was attributed to the stickiness of the powder. During the spray-drying experiments and FSI trials, it was observed that the RIF-ETH SD was very electrostatic and sticky. The stickiness could be a result of a not-completely dry or an extremely dry powder. This has to be optimized in further spray-drying tests (e.g., spray-drying conditions, addition of excipients). Especially in comparison to the PM but also to the SD formulation, the co-milled COAMS delivered much more reproducible results, which is reflected in the low standard deviation of ED and FPF values. The deviations are higher for RIF compared to ETH; for ETH, FPF and ED, the standard deviations are not that high, so it is concluded that the ETH is distributed more homogenously within the formulations. However, overall, the delivery is still much more constant for the milled COAMS compared to the PM. This is in further agreement with results from mixing homogeneity (Table 3), which were much higher for the PM than for the co-amorphous formulations. This shows that the administration of COAMSs can reduce drug-dose variability when administering combination products.

**Figure 4:**
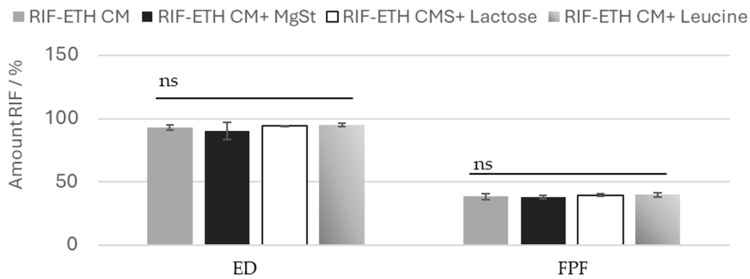
EF and FPF or RIF for ETH-RIF CM with the addition of MgSt, leucine or lactose, (mean ± SD, *n* = 3), ns: not significant.

Typically, carrier-free formulations use advanced technologies like spray-drying or special technologies (Pulmospheres^®^, Technospheres^®^ etc.) to yield fine particles with adequate flowability and good dispersion properties, eliminating the need for a carrier material [30,47]. Another way to improve formulation properties of carrier free formulations and facilitate drug dispersion is the addition of fine pharmaceutical excipients. Therefore, for the most promising formulation (RIF-ETH CM), the addition of fine excipients was tested to check whether the performance can even be improved. Common excipients used in DPI products were used, namely MgSt (0.5%), leucine (5%) and fine lactose (10%). Results showed that the addition of fine excipients does not significantly change the aerosolization properties. The ED and the FPF are comparable between the COAMS without and with excipients (Figure 4). Also, the standard deviations are low, so deviations are very low. So, we can conclude that for the milled COAMS, the addition of ternary agents in the typical concentration applied does not have a beneficial effect.

It is worth mentioning that without formulation optimization, an FPF of 38%, as compared to commercial DPI products on the market, is rather high. Typical DPIs on the market show FPFs of around 20–30% [48]. Often formulation and inhaler devices are also co-developed in order to optimize and maximize performance. So here there also lies potential to improve the performance. Further spray-drying of RIF-ETH COAMS has the potential to be optimized further (use of excipients and adaptation of process parameters) to optimize powder properties for higher aerosolization performance.

**Figure 5:**
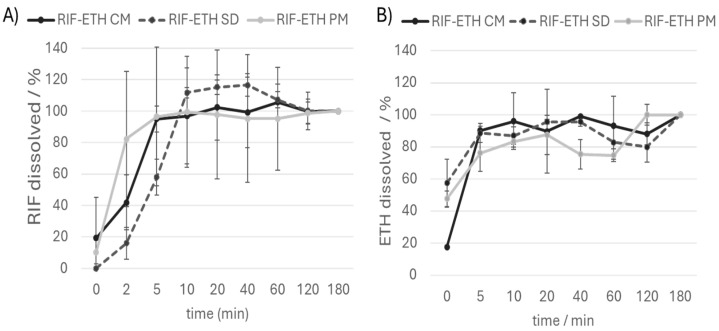
Dissolution curve (amount of (**A**) RIF and (**B**) ETH dissolved over time) of the RIF ETH physical mixture (RIF ETH PM), the cryo-milled formulation (RIF-ETH CM) and the spray-dried formulation (RIF-ETH SD), (mean ± SD, *n* = 3). Values are referred to the dissolved drug amount after 180 min. That amount is set at 100%.

Advantages associated with the amorphous form of a drug as well as COAMSs are the higher solubility, faster dissolution and potential higher bioavailability [49]. To assess whether the administration of COAMSs is also beneficial in this context, dissolution studies in SLF were performed for the RIF-ETH PM and the COAMS of RIF-ETH (Figure 5).

Comparing the dissolution curves of the three formulations, no striking differences were noted. It is noticeable that all three formulations reach a plateau after about 10 min. Values above 100% might be due to sampling issues (sample taken too close to the filter; this can lead to localized supersaturation or an artificially high concentration due to undissolved or partially dissolved particles) or incomplete mixing of the media. In contrast to what was expected, co-amorphous systems did not dissolve faster than the PM. A similar finding was obtained in spray-dried co-amorphous systems of cilostazol and L-tryptophan [50]. One possible explanation for this behavior is that a larger surface area is exposed in the PM. From SEM images (Figure 2A1,A2), the SD (standard deviation) of the drug content (Table 3) and the variation in the FPF (Table 4), it can be seen that, despite micronization, the composition of the PM was not homogenous. This is also the most likely reason why the PM shows a very large fluctuation in its dissolution behavior (recognizable by very high standard deviations and error bars). RIF-ETH COAMS show much more reproducible results compared to the PM, with fewer deviations in terms of FPF and drug quantity dissolved over time.

## 4. Conclusions

Our ML tool was able to predict drug combinations for co-amorphous systems that could be developed into a stable co-amorphous formulation with favorable aerosolization properties. This means that the program can accelerate the development of new formulations by selecting the suitable drug combinations. It is not expected that the tool will be able to help you choose the process of particle generation. However, based on the predictions of our model, a successful co-amorphous formulation of RIF-ETH, suitable for inhalation, was developed with either spray-drying or cryo-milling. Compared to a pure physical mixture of crystalline RIF-ETH, the milled RIF ETH COAMS in particular delivered more constant APIs (smaller standard deviations and higher EF) to the lung. Since the addition of fine pharmaceutical excipients did not improve performance, it would be interesting to find out if the tool is able to differentiate between drug combinations that require pharmaceutical excipients and others that do not benefit from the addition of excipients. This could provide an added benefit when using the tool.

## Figures and Tables

**Table 1:** List of API-API combinations tested experimentally; the related prediction and distance from the training data, based on the predictive ML model; the therapeutic relevance of the API combinations; the results from XRD measurements (indicating if both or only one of the APIs are amorphous or crystalline); and validation success (yes—agreement with prediction; no—agreement with prediction).

API 1	API 2	Prediction	Distance	Therapeutic Relevance	XRPD Results	Validation Success
MOM	BAM	1	57	IGC + LAMA	amorphous both	yes
SBS	RIF	1	184		amorphous both	yes
RIF	GB	1	253		amorphous both	yes
GB	STR	1	411		amorphous both	yes
RIF	ETH	1	361	Two first-line drugs	amorphous both	yes
MOM	SBS	1	117		amorphous both	yes
PYR	ISO	1	55	Two first-line drugs	crystalline both	no
MOM	GB	0.98	58	IGC + LABA	amorphous both	yes
RIF	PYR	0.98	394	Two first-line drugs	amorphous both	yes
BUD	GB	0.98	74	IGC + LABA	amorphous both	yes
ETH	PYR	0.96	44	Two first-line drugs	crystalline both	no
SBS	GB	0.94	90		amorphous both	yes
ETH	ISO	0.9	57	Two first-line drugs	crystalline both	no
BAM	PYR	0.26	78		crystalline both	yes
BAM	ISO	0.22	65		crystalline both	yes
BUD	STR	0.14	187		amorphous STR	no
MOM	STR	0.12	212		amorphous STR	no
ETH	GB	0	89		crystalline both	yes

IGC—inhalable glucocorticoid, LABA—long-acting beta receptor agonist, LAMA—long-acting muscarinic receptor antagonist, MOM—mometasone, BAM—bambuterol HCl, SBS—salbutamol sulfate, RIF—rifampicin, GB—glycopyrronium bromide, STR—streptomycin sulfate, ETH—ethambutol dihydrochloride, ISO—isoniazid, PYR—pyrazinamide, BUD—budesonide.

**Table 2:** Particle size (D10, D50 and D90 (all forward scatter)) of the starting materials (jet milled (JM) RIF and ETH), the physical mixture of RIF and ETH (RIF-ETH PM) and the co-amorphous milled (RIF-ETH CM) and spray-dried (RIF-ETH SD) RIF-ETH formulation (mean ± SD, *n* = 3).

Formulation	D10 Intensity/µm	D50 Intensity/µm	D90 Intensity/µm	Undersize SPAN
RIF JM	0.91 ± 0.51	1.49 ± 1.08	2.54 ± 1.30	1.08 ± 0.79
ETH JM	0.85 ± 0.46	1.38 ± 0.83	2.40 ± 1.23	2.01 ± 0.27
RIF + ETH PM	1.61 ± 0.48	2.13 ± 0.64	2.87 ± 0.82	0.94 ± 0.70
RIF + ETH CM	2.02 ± 0.48	2.78 ± 0.95	3.83 ± 1.04	0.71 ± 0.50
RIF + ETH SD	1.59 ± 0.50	2.01 ± 0.65	2.84 ± 0.86	0.85 ± 0.74

**Table 3:** Mean drug content (%) and mixing homogeneity (relative standard deviation of mean drug content (%)) for RIF and ETH within the 3 formulations RIF-ETH PM, RIF-ETH CM and RIF-ETH CM (*n* = 10).

Formulation	Mean Drug Content RIF/%	Mixing Homogeneity RIF/%	Mean Drug Content ETH/%	Mixing Homogeneity ETH/%
RIF + ETH PM	104.7	6.0	81.2	10.2
RIF + ETH CM	96.8	4.5	96.2	1.6
RIF + ETH SD	96.1	2.3	92.0	3.6

**Table 4:** Aerodynamic assessment results of rifampicin for the physical mixture of RIF and ETH (RIF-ETH PM), and the co-amorphous milled (RIF-ETH CM) and spray-dried (RIF-ETH SD) formulation (mean ± SD, *n* = 3).

Formulation	EF RIF/%	FPM RIF/µg	FPF RIF/%
RIF + ETH PM	72.7 ± 28.2	6291.3 ± 1522.0	48.78 ± 13.1
RIF + ETH CM	93.1 ± 2.0	5450.2 ± 214.2	38.54 ± 2.3
RIF + ETH SD	71.0 ± 11.8	3592.12 ± 705.8	32.38 ± 2.8

**Table 5:** Aerodynamic assessment results of ETH for the physical mixture of RIF and ETH (RIF-ETH PM), the co-amorphous milled (RIF-ETH CM) sample and spray-dried (RIF-ETH SD) formulation (mean ± SD, *n* = 3).

Formulation	EF ETH/%	FPM ETH/µg	FPF ETH/%
RIF + ETH PM	90.2 ± 0.5	3097.8 ± 210.4	40.46 ± 1.4
RIF + ETH CM	95.9 ± 0.6	3810.8 ± 224.4	40.20 ± 0.2
RIF + ETH SD	66.2 ± 14.9	1713.0 ± 629.8	38.27 ± 14.3

## Data Availability

The original contributions presented in this study are included in the article/Supplementary Material. Further inquiries can be directed to the corresponding author.

## Supplementary Materials

The following supporting information can be downloaded at https://www.mdpi.com/article/10.3390/pharmaceutics17070922/s1. Table S1: Selection of molecular descriptors; Figure S1: XRD graphs of API-API combinations after co-milling for model validation. Each graph shows one co-milled combination and the respective starting APIs of (A) MOM-BAM, (B) SBS-RIF, (C) RIF-GB, (D) GB-STR, (E) RIF-ETH, (F) MOM-SBS, (G) PYR-ISO, (H) MOM-GB, (I) RIF-PYR, (J) BUD-GB, (K) ETH-PYR, (L) SBS-GB, (M) ETH-ISO, (N) BAM-PYR, (O) BAM-ISO, (P) BUD-STR, (Q) MOM-STR and (R) ETH-GB; (MOM—mometasone, BAM—bambuterol HCl, SBS—salbutamol sulfate, RIF—rifampicin, GB—glycopyrronium bromide, STR—streptomycin sulfate, ETH—ethambutol dihydrochloride, ISO—isoniazid, PYR—pyrazinamide, BUD—budesonide); Figure S2: SEM images of rifampicin starting material (A1), rifampicin micronized (A2) and ethambutol starting material (B1) and ethambutol micronized (B2).; Figure S3: XRD pattern of the (A) co-amorphous milled (RIF-ETH CM) RIF-ETH formulation and (B) spray-dried (RIF-ETH SD) RIF-ETH formulation after 365 days.
