# Supplementary material for: Development of Co-Amorphous Systems for Inhalation Therapy—Part 1: From Model Prediction to Clinical Success
Source: Pharmaceutics. 2025 Jul 16;17(7):922. doi: 10.3390/pharmaceutics17070922 (PMC12299485; doi:10.3390/pharmaceutics17070922)
Supplement: Supplementary file 1 [file pharmaceutics-17-00922-s001.zip › pharmaceutics-3634810-supplementary.pdf]

pharmaceutics

MDPI

Development of Co-Amorphous Systems for Inhalation Therapy—Part 1: from Model Prediction to Clinical Success

Eleonore Fröhlich, Aurora Bordoni, Nila Mohsenzada, Stefan Mitsche and Hartmuth Schröttner and Sarah Zellnitz-Neugebauer

Table S1. Selected molecular descriptors (Fink et al., 2023.)

Molecular Descriptor Abbreviation	Molecular Descriptor Explanation	Molecular Descriptor Abbreviation	Molecular Descriptor Explanation	Molecular Descriptor Abbreviation	Molecular Descriptor Explanation
**ABC**	atom bond connectivity index	**Diameter**	topological diameter	**RNCS**	relative negative charge surface area
**nAcid**	acidic group count	**TopoShapeIndex**	topological shape index	**RPCS**	relative positive charge surface area
**nBase**	basic group count	**nRot**	rotatable bonds count	**TASA**	total hydrophobic surface area
**nAromAtom**	aromatic atoms count	**SLogP**	Wildman-Crippen log P	**TPSA**	total polar surface area
**nAromBond**	aromatic bond count	**TopoPSA**	topological polar surface area	**RASA**	relative hydrophobic surface area
**nAtom**	number of all atoms	**naRing**	aromatic ring count	**RPSA**	relative polar surface area
**nHeavyAtom**	number of heavy atoms	**apol**	atomic polarisability	**fMF**	molecular framework ratio
**nHetero**	number of hetero atoms	**bpol**	bond polarisability	**Vabc**	ABC van der Waals volume
**nH**	number of H atoms	**nHBAcc**	number of hydrogen bond acceptor	**VAdjMat**	vertex adjacency information
**MW**	molecular weight	**nHBDon**	number of hydrogen bond donors		

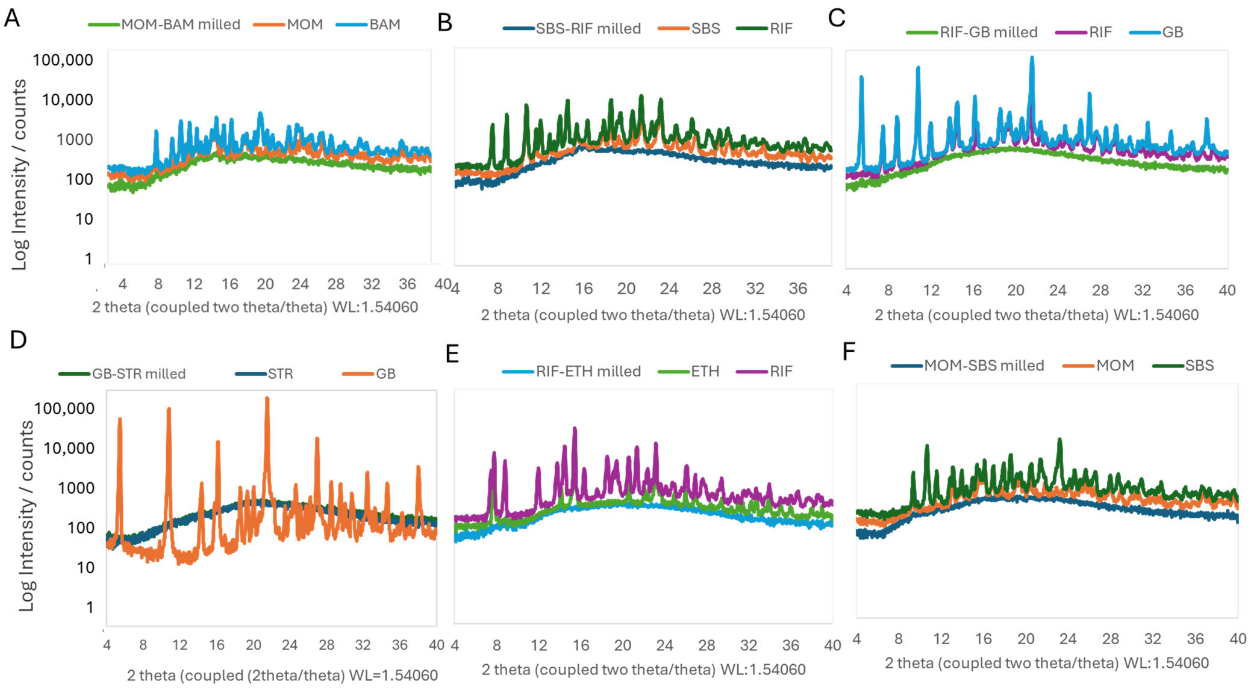
A
MOM-BAM milled
MOM
BAM
B
SBS-RIF milled
SBS
RIF
C
RIF-GB milled
RIF
GB
D
GB-STR milled
STR
GB
E
RIF-ETH milled
ETH
RIF
F
MOM-SBS milled
MOM
SBS
Log Intensity / counts
100,000
10,000
1000
100
10
1
4 8 12 16 20 24 28 32 36 40
2 theta (coupled two theta/theta) WL:1.54060
2 theta (coupled (2theta/theta) WL=1.54060

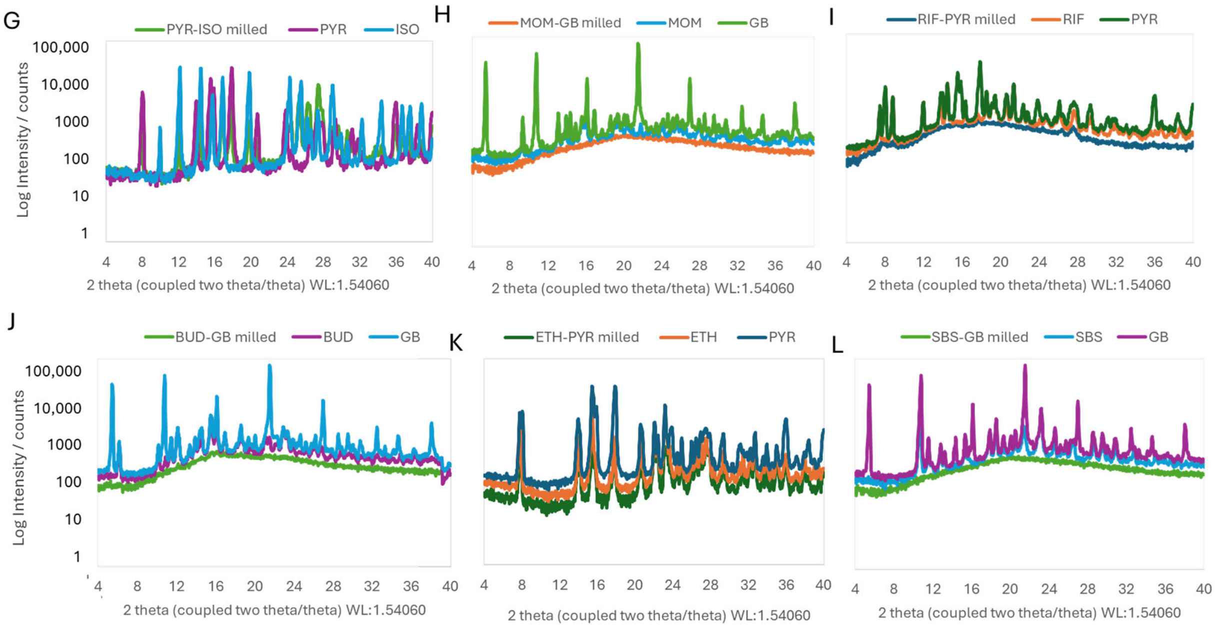
G
PYR-ISO milled
PYR
ISO
H
MOM-GB milled
MOM
GB
I
RIF-PYR milled
RIF
PYR
J
BUD-GB milled
BUD
GB
K
ETH-PYR milled
ETH
PYR
L
SBS-GB milled
SBS
GB
Log Intensity / counts
100,000
10,000
1000
100
10
1
4 8 12 16 20 24 28 32 36 40
2 theta (coupled two theta/theta) WL:1.54060

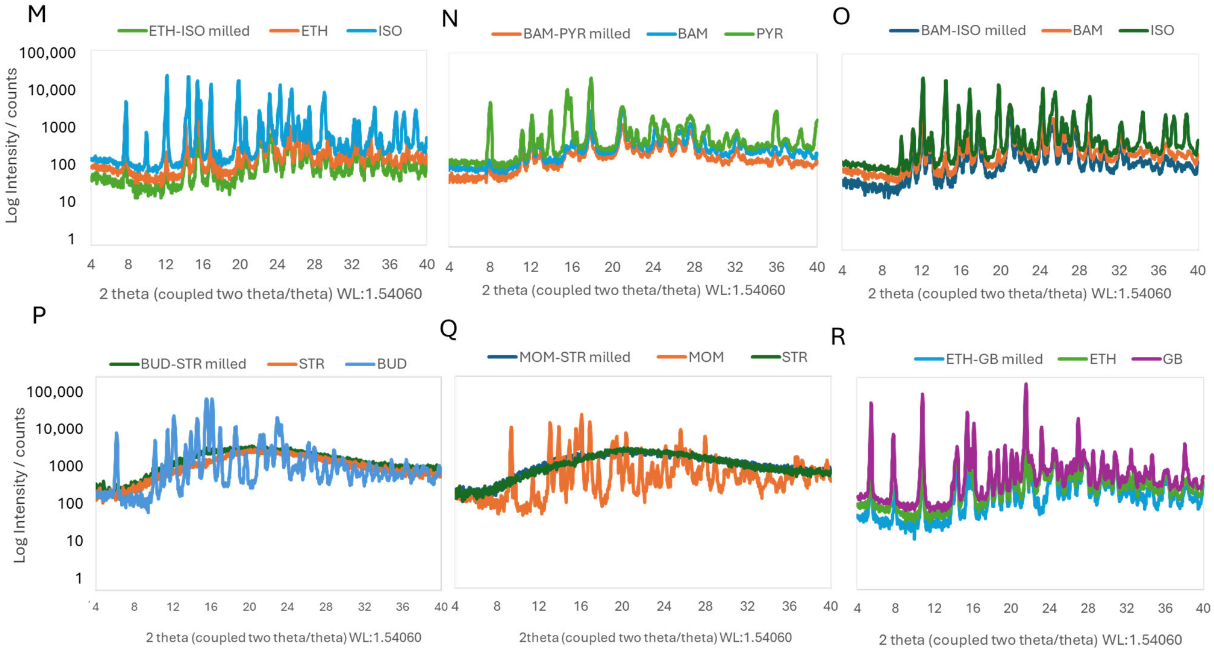

Figure S1. XRD graphs of API-API combinations after co-milling for model validation. Each Graph shows one co-milled combination and the respective starting APIs of **A**) MOM-BAM, **B**) SBS-RIF, **C**) RIF-GB, **D**) GB-STR, **E**) RIF-ETH, **F**) MOM-SBS, **G**) PYR-ISO, **H**) MOM-GB, **I**) RIF-PYR, **J**) BUD-GB, **K**) ETH-PYR, **L**) SBS-GB, **M**) ETH-ISO, **N**) BAM-PYR, **O**) BAM-ISO, **P**)BUD-STR, **Q**) MOM-STR and **R**) ETH-GB; (MOM – mometasone, BAM – bambuterol HCl, SBS – salbutamol sulphate, RIF – rifampicin, GB – glycopyrronium bromide, STR – streptomycin sulphate, ETH – ethambutol dihydrochloride, ISO – isoniazide, PYR – pyrazinamide, BUD – budesonide).

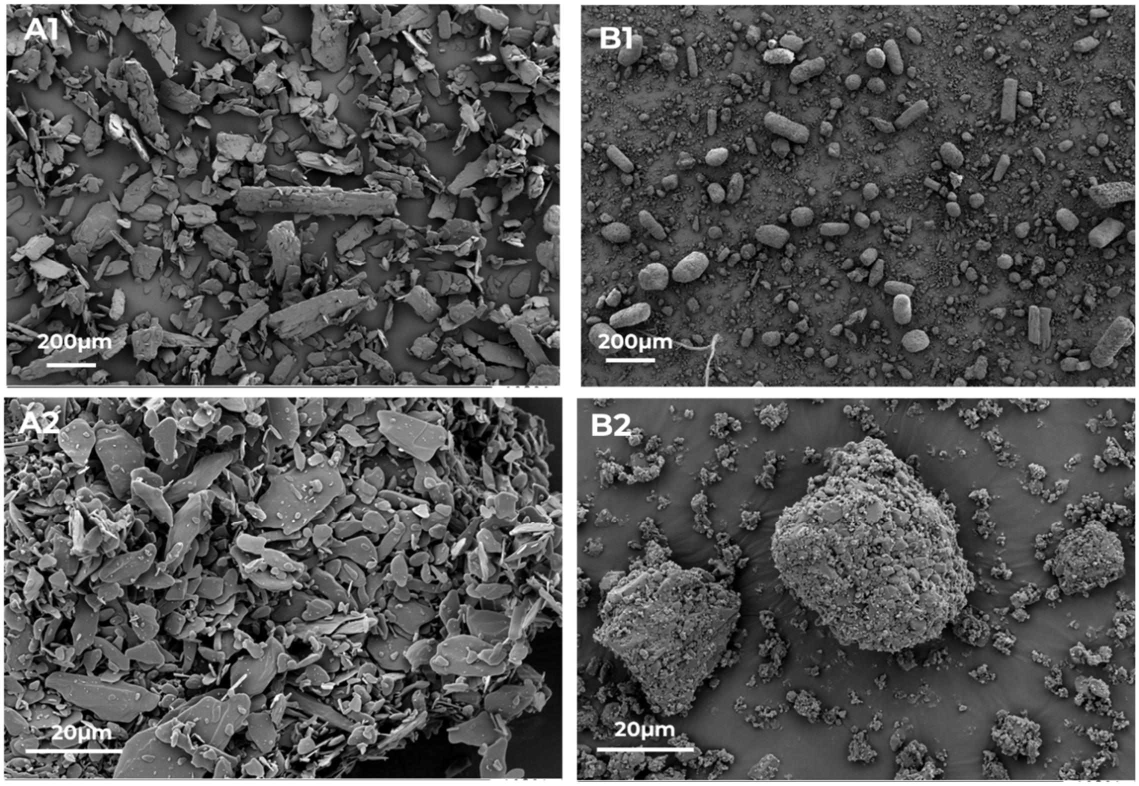

Figure S2. SEM images of Rifampicin starting material (**A1**), Rifampicin micronized (**A2**) and Ethambutol starting material (**B1**) and Ethambutol micronized (**B2**).

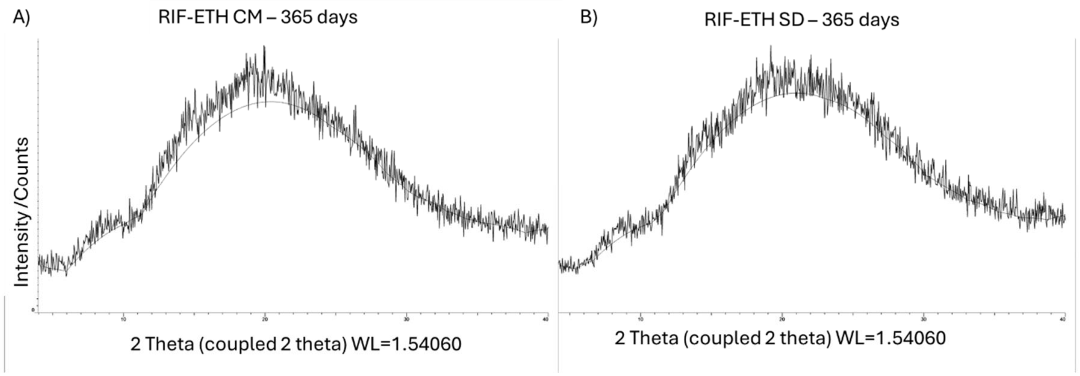

Figure S3. XRD pattern of the **A**) co-amorphous milled RIF-ETH formulation (RIF-ETH CM) and **B**) spray-dried RIF-ETH formulation (RIF-ETH SD) after 365 days.

Reference

Fink, E.; Brunsteiner, M.; Mitsche, S.; Schröttner, H.; Paudel, A.; Zellnitz-Neugebauer, S. Data-Driven Prediction of the Formation of Co-Amorphous Systems. Pharmaceutics **2023**, *15*, 347, doi:10.3390/pharmaceutics15020347.
